# Biomedical-grade, high mannuronic acid content (BioMVM) alginate enhances the proteoglycan production of primary human meniscal fibrochondrocytes in a 3-D microenvironment

**DOI:** 10.1038/srep28170

**Published:** 2016-06-15

**Authors:** Ana Rey-Rico, Angelique Klich, Magali Cucchiarini, Henning Madry

**Affiliations:** 1 Center of Experimental Orthopaedics, Saarland University, D-66421 Homburg, Germany; 2 Department of Orthopaedic Surgery, Saarland University Medical Center, Saarland University, D-66421 Homburg, Germany

## Abstract

Alginates are important hydrogels for meniscus tissue engineering as they support the meniscal fibrochondrocyte phenotype and proteoglycan production, the extracellular matrix (ECM) component chiefly responsible for its viscoelastic properties. Here, we systematically evaluated four biomedical- and two nonbiomedical-grade alginates for their capacity to provide the best three-dimensional (3-D) microenvironment and to support proteoglycan synthesis of encapsulated human meniscal fibrochondrocytes *in vitro*. Biomedical-grade, high mannuronic acid alginate spheres (BioLVM, BioMVM) were the most uniform in size, indicating an effect of the purity of alginate on the shape of the spheres. Interestingly, the purity of alginates did not affect cell viability. Of note, only fibrochondrocytes encapsulated in BioMVM alginate produced and retained significant amounts of proteoglycans. Following transplantation in an explant culture model, the alginate spheres containing fibrochondrocytes remained in close proximity with the meniscal tissue adjacent to the defect. The results reveal a promising role of BioMVM alginate to enhance the proteoglycan production of primary human meniscal fibrochondrocytes in a 3-D hydrogel microenvironment. These findings have significant implications for cell-based translational studies aiming at restoring lost meniscal tissue in regions containing high amounts of proteoglycans.

Meniscal lesions are frequent, have a reduced ability to heal^1^, and may induce osteoarthritis^2^. Tissue engineering approaches are of high interest to repair or replace damaged meniscus tissue^3^,^4^,^5^,^6^,^7^,^8^,^9^. Scaffolds for meniscus tissue engineering include hydrogels, synthetic polymers, and tissue-derived materials^10^. An ideal scaffold may maintain the cellular phenotype, induce extracellular matrix (ECM) synthesis^10^, and be biocompatible and biodegradable with a degradation rate adapted to the remodelling process^1^,^10^,^11^. Hydrogels are especially advantageous^1^,^12^,^13^,^14^ compared with solid matrices, as they provide a three-dimensional (3-D) specific microenvironment and retain significant amounts of incompressible fluids^1^. Their ability to reversibly form gels in response to environmental factors is an additional potential advantage^15^. Alginate is the most commonly employed hydrogel matrix to immobilize cells including meniscal fibrochondrocytes, chondrocytes, myoblasts, mesenchymal and embryonic stem cells, pancreatic islet cells, hepatocytes, parathyroid cells, and liver cells both *in vitro* and *in vivo*^12^,^16^,^17^,^18^,^19^,^20^,^21^,^22^,^23^,^24^.

The composition of alginate directly influences, among other factors^25^, the physiochemical properties of the hydrogel meshwork^26^ and consequently the behavior of the encapsulated cells^27^,^28^. For example, biomedical-grade, low-viscosity, high-guluronic acid content alginate provides an optimal 3-D microenvironment for articular chondrocyte proliferation, while biomedical-grade, medium-viscosity, high-mannuronic acid content alginates promote the production of proteoglycans^27^,^28^. Little is known, however, on the impact of composition of this 3-D microenvironment on the metabolic activities of meniscal fibrochondrocytes. Verdonk and co-workers showed that, compared with monolayer culture condition, meniscal cells had a more fibrochondrocyte-like cell phenotype when embedded in a nonbiomedical-grade alginate^29^. As proteoglycans are the ECM component that chiefly determines the viscoelastic compressive properties of a meniscus^30^, the effect on the proteoglycan production of the alginate composition is another key factor. However, a methodical evaluation of the effect of alginate composition, viscosity, and purity on the proteoglycan production and capacity to maintain the phenotype of meniscal fibrochondrocytes has, to our best knowledge, not been performed to date.

Here, we systematically evaluated four biomedical- and two nonbiomedical-grade alginates for their capacity to support the *in vitro* culture of human meniscal fibrochondrocytes. We specifically hypothesized that the composition (high mannuronic acid *versus* high guluronic acid), viscosity (low *versus* medium viscosity), and degree of purity of alginates (nonbiomedical- *versus* biomedical-grade alginates) directly influence the morphology of the spheres and the viability, proliferation, and proteoglycan synthesis of encapsulated human meniscal fibrochondrocytes *in vitro*. In addition, an experimental model of the transplantation of such spheres containing meniscal fibrochondrocytes into a human meniscal defect *in vitro* is proposed with the view of testing the suitability of hydrogel spheres for future translational approaches.

## Results

### Morphological characterization of alginate spheres containing human meniscal fibrochondrocytes

**Figure 1:**
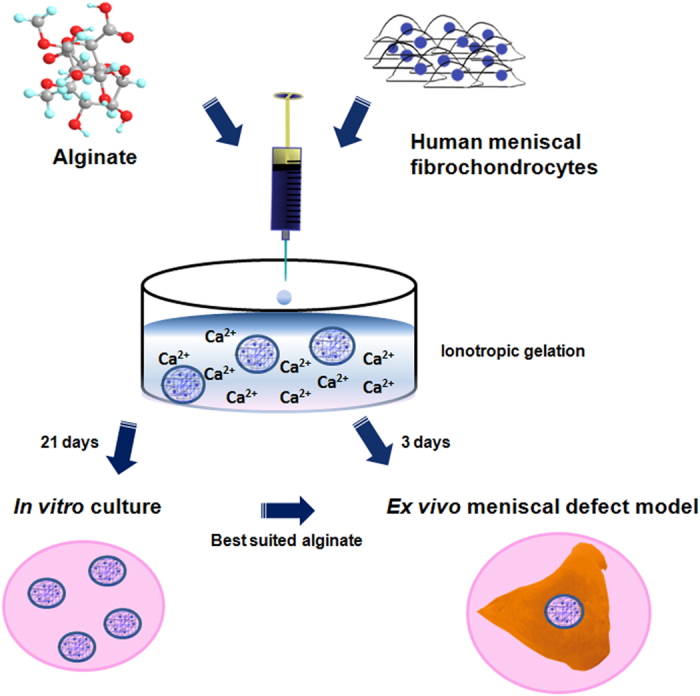
Experimental design. Human meniscal fibrochondrocytes were resuspended in four types of biomedical- and two types of nonbiomedical-grade alginates and extruded through a 21-gauge needle into a solution of 102 mM CaCl_2_. Cell-hydrogel constructs formed from the different types of alginates were cultured for 21 days *in vitro*. The best suited alginate providing a favorable 3-D microenvironment to support the proteoglycans production by human meniscal fibrochondrocytes was tested in an experimental model of meniscal defect *ex vivo*.

**Figure 2:**
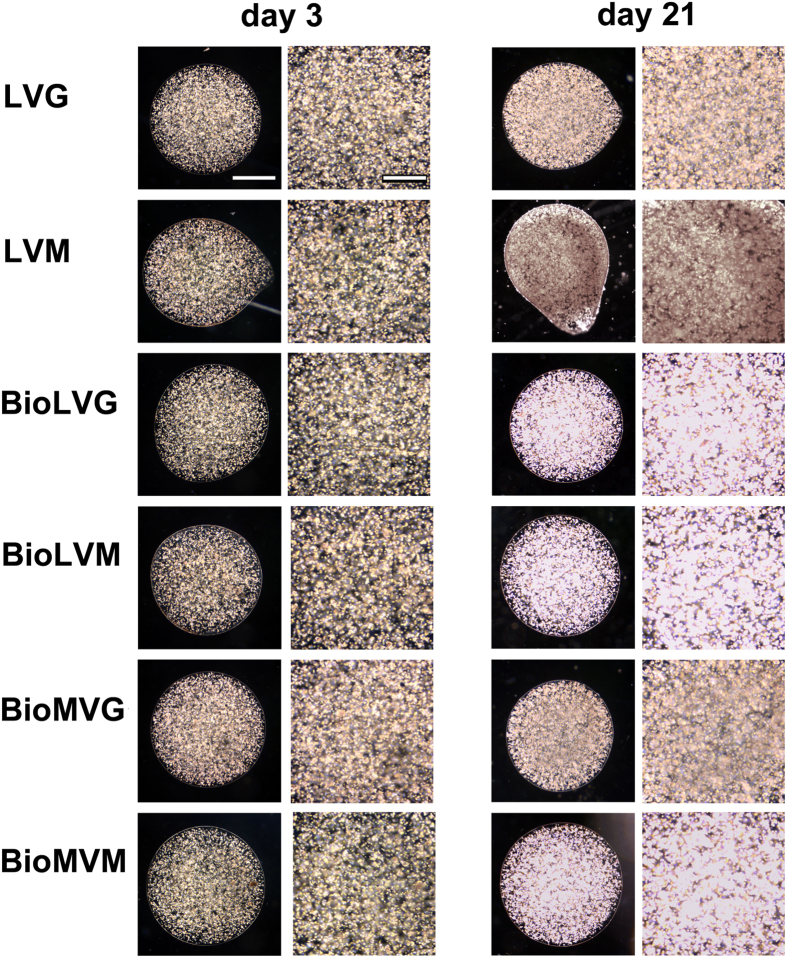
Photomicrographs of spheres obtained from the LVG and LVM nonbiomedical-grade alginates and the BioLVG, BioLVM, BioMVG, and BioMVM biomedical-grade alginates (scale bar 1,000 μm).

**Figure 3:**
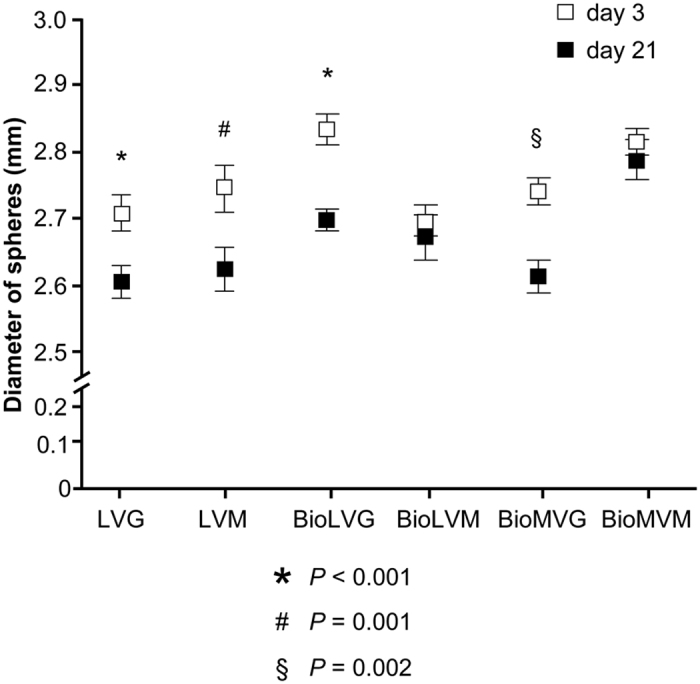
Mean diameter of alginate spheres with human meniscus cells on day 3 and day 21 (*, #, and § indicate *P* < 0.001, *P* = 0.001, and *P* = 0.001, respectively, compared with day 3).

Human meniscal fibrochondrocytes were first encapsulated in various alginates as designed in Fig. 1 to determine the best suited compound that provides a favorable 3-D microenvironment for proteoglycan synthesis based on an evaluation of the morphology of the resulting spheres over an extended period of time. Application of biomedical-grade alginates always resulted in spheres that were spherically more uniform and could be prepared with a greater reproducibility compared with nonbiomedical-grade alginates (Fig. 2). The mean sphere diameters ranged between 2.69 ± 0.02 mm (BioLVM), 2.71 ± 0.03 mm (LVG), 2.74 ± 0.02 mm (BioMVG), 2.74 ± 0.03 mm (LVM), 2.81 ± 0.02 mm (BioMVM), and 2.83 ± 0.02 mm (BioLVG). Spheres composed of BioLVM displayed the smallest diameter, significantly different than those of spheres made of BioLVG (*P* = 0.001), BioMVM (*P* < 0.001), or BioMVG (*P* = 0.007). The diameters of all other spheres were not significantly different on day 3 (*P* > 0.05). After 21 days *in vitro*, the diameters of the LVG, LVM, BioLVG, and BioMVG spheres significantly decreased, with BioLVG and LVG spheres showing the most pronounced decrease (4.8% and 3.8%, respectively) (*P* < 0.001). There was no significant difference in the diameters of the BioLVM and BioMVM spheres over time (*P* > 0.05) (Fig. 3).

**Figure 4:**
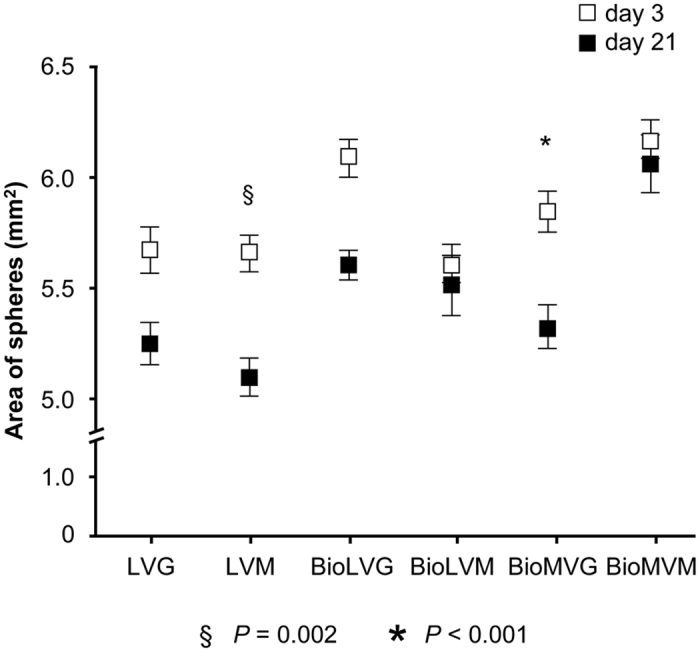
Area of alginate spheres with human meniscal fibrochondrocytes on day 3 and day 21. (* and § indicate *P* < 0.001 and *P* = 0.002, respectively, compared with day 3).

The mean area of the spheres was of 5.61 ± 0.09 mm^2^ (BioLVM), 5.66 ± 0.08 mm^2^ (LVM), 5.67 ± 0.12 mm^2^ (LVG), 5.84 ± 0.09 mm^2^ (BioMVG), 6.08 ± 0.09 mm^2^ (BioLVG), and 6.17 ± 0.09 mm^2^ (BioMVM). Spheres composed of BioLVM exhibited the lowest area that was significantly different from that of spheres made of BioMVG (*P* = 0.003), BioLVG (*P* < 0.001), and BioMVM (*P* < 0.001). No differences were observed for all other spheres on day 3 (*P* > 0.05). After 21 days *in vitro*, the area of all spheres decreased, with BioMVG and LVM spheres having the most pronounced decrease (8.7% and 9.9%, respectively) (*P* ≤ 0.002) while the BioLVM and BioMVM spheres showed only minor changes (*P* > 0.05) (Fig. 4).

### Morphology, viability, and proliferation of human meniscal fibrochondrocytes in alginate spheres

**Figure 5:**
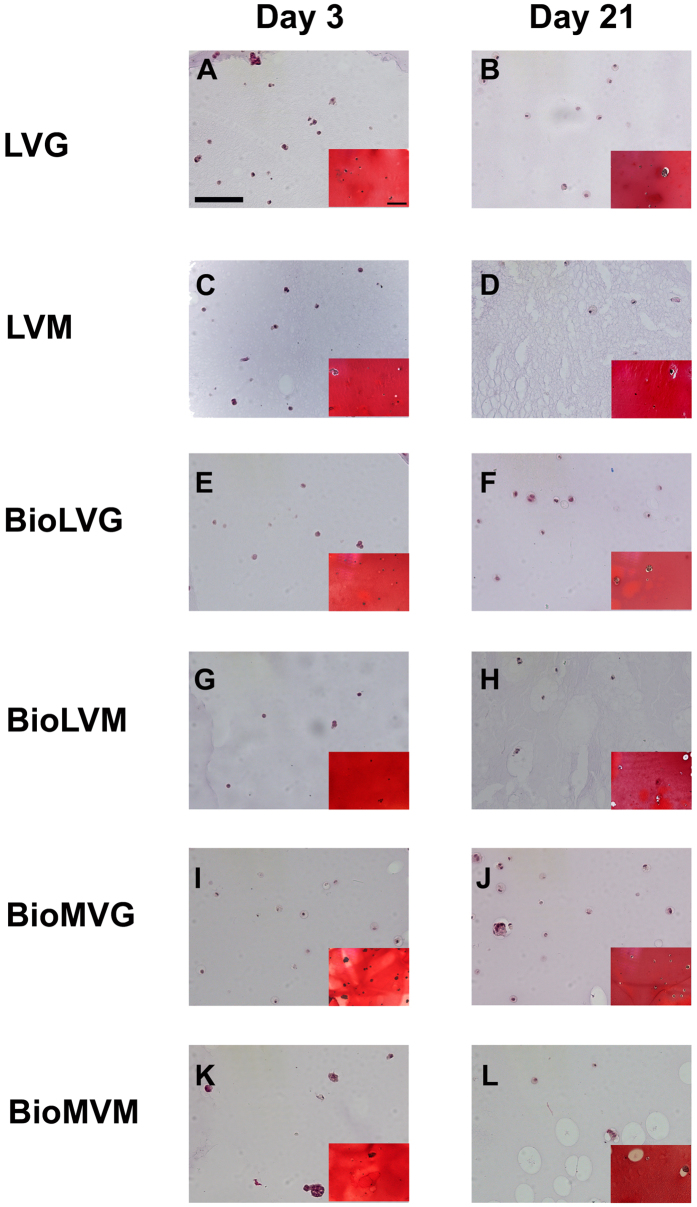
Histological analysis of H&E and Safranin O (insets) representative sections of primary human meniscal fibrochondrocytes encapsulated in alginate spheres made of LVG (**A,B**), LVM (**C,D**), BioLVG (**E,F**), BioLVM (**G,H**), BioMVG (**I,J**), and BioMVM (**K,L**). Scale bar 100 μm.

Histological evaluation of human meniscal fibrochondrocytes embedded in the different alginate spheres on day 3 after encapsulation mainly revealed cells with an oval or round morphology and were homogenously distributed without detectable differences between groups (Fig. 5).

**Figure 6:**
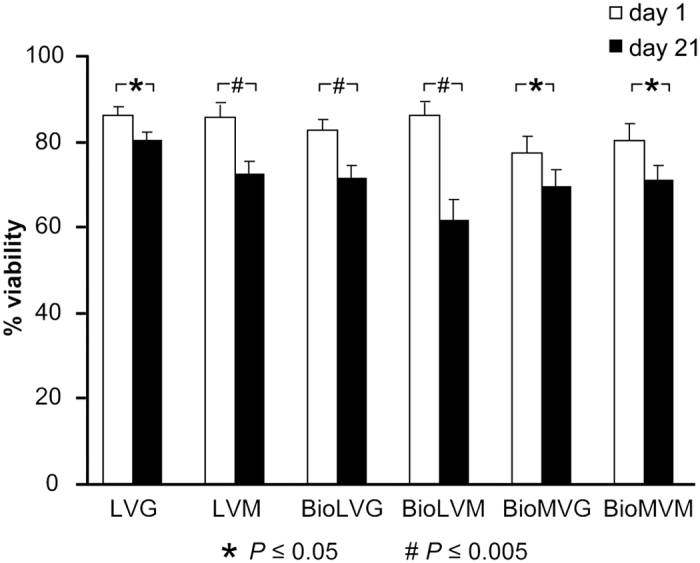
Viability of human meniscal fibrochondrocytes embedded in LVG, LVM, BioLVG, BioLVM, BioMVG, and BioMVM alginate on day 1 and 21 (* and # indicate *P* ≤ 0.05 and *P* ≤ 0.005, respectively).

On day 1 post-encapsulation, cell viability ranged between 80.4% and 86.0% (*P* > 0.05). After 21 days post-encapsulation, human meniscal fibrochondrocytes encapsulated in LVG showed the highest levels of viability (~80%), significantly higher than cells encapsulated in BioLVM (*P* < 0.001) or in LVM, BioLVG, BioMVG, and BioMVM (*P* < 0.05), with values ranging from 61.7% to 80.3%, respectively (Fig. 6). The cell viabilities always decreased over time. The highest decline was observed using BioLVM alginate (28.3% decrease compared with day 3; *P* < 0.001), while cell viability only slightly decreased in LVG alginate (6.6%, *P* < 0.05).

**Figure 7:**
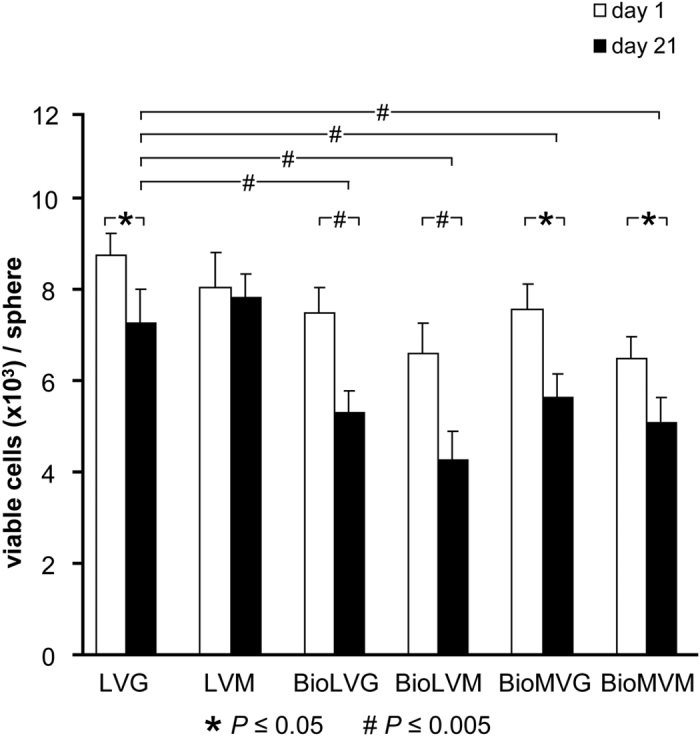
Number of viable human meniscal fibrochondrocytes embedded in LVG, LVM, BioLVG, BioLVM, BioMVG, and BioMVM alginate on day 1 and 21.(* and # indicate *P* ≤ 0.05 and *P* ≤ 0.005, respectively).

**Figure 8:**
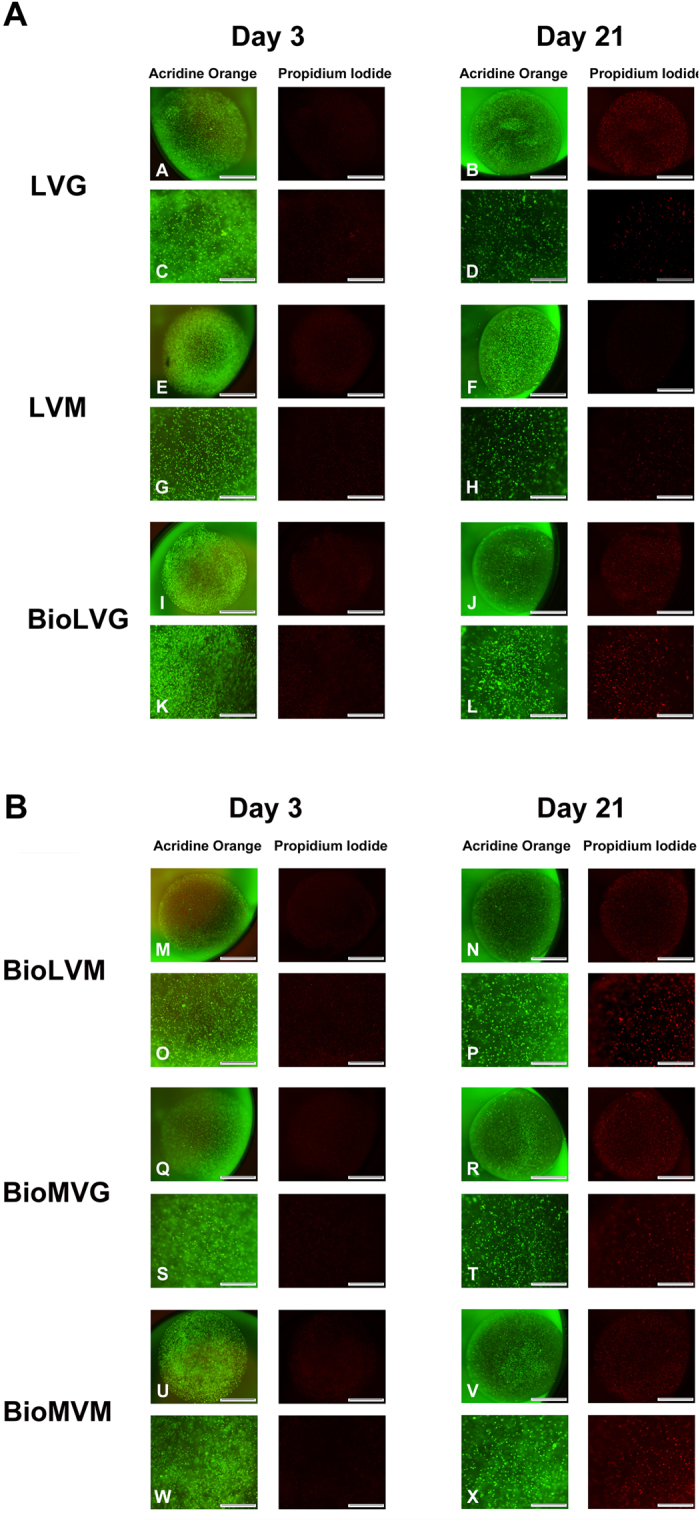
Viability of human meniscal fibrochondrocytes after encapsulation in alginate spheres at 3 and 21 days post-encapsulation in (**A**). LVG (A–D), LVM (E–H), and BioLVG (I–L) and in (**B**). BioLVM (M–P), BioMVG (Q–T), and BioMVM (U–X). Cells were stained with acridine orange (living cells, in green) and propidium iodide (dead cells, in red). Scale bars 1,000 μm (A-B, E-F, I-J, M-N, Q-R, and U-V) and 500 μm (C-D, G-H, K-L, O-P, S-T, and W-X).

On day 1 post-encapsulation, all spheres contained 7.48 ± 0.58 × 10^3^ viable human meniscal fibrochondrocytes (Fig. 7). After 3 weeks of culture, the numbers of viable cells significantly decreased in all alginates except for LVM. The highest decline in the numbers of viable cells was seen upon encapsulation in BioLVM and BioLVG alginate (35.2% and 29.2%, respectively). The LVG and LVM spheres contained the highest numbers of viable cells at this time point. Live/dead staining of entire spheres containing human meniscal fibrochondrocytes revealed a similar pattern (Fig. 8). After 3 days post-encapsulation, a high number of human meniscal fibrochondrocytes was viable in all types of alginates (green fluorescence), while only a reduced number of cells was dead (red fluorescence) (Fig. 8). After 3 weeks, the highest amount of dead cells was stained when using BioLVM and BioLVG, in contrast to LVG or LVM spheres that showed the lowest amount of red fluorescence signal.

### DNA and proteoglycan contents of alginate spheres containing human meniscal fibrochondrocytes

**Figure 9:**
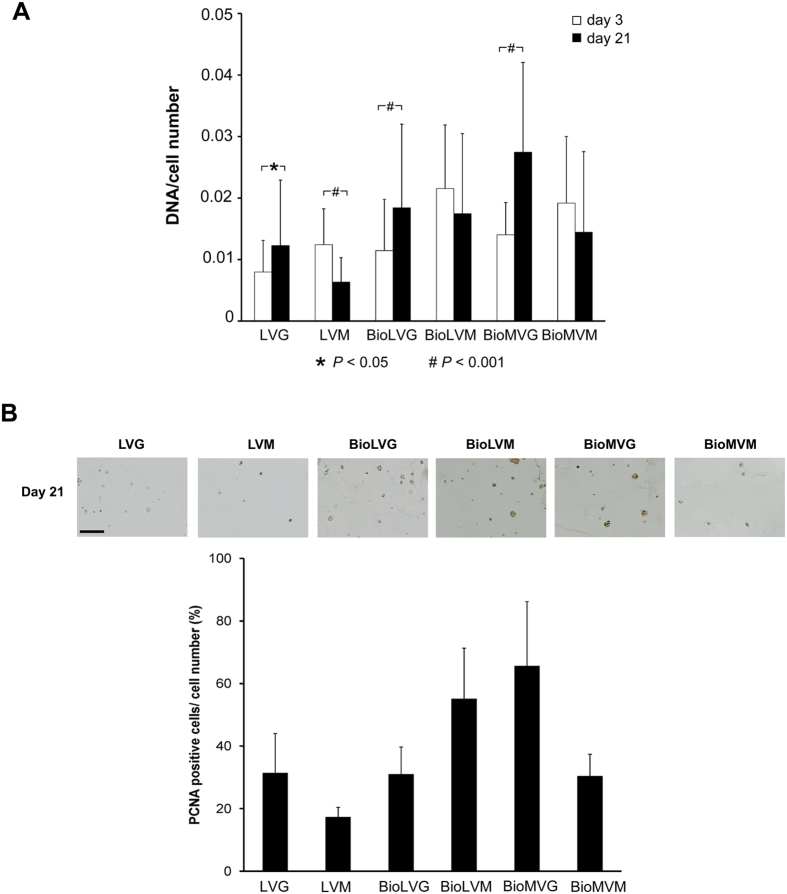
(**A**) DNA contents of human meniscal fibrochondrocytes encapsulated in alginate spheres standardized to the cell numbers on day 3 and day 21 (* indicates *P* ≤ 0.001). (**B**) Immunohistochemical and histomorphometrical analyses of PCNA-positive cells on day 21 (magnification 20x, scale bar 100 μm).

The DNA contents of human meniscal fibrochondrocytes encapsulated in the different constructs were estimated after 3 and 21 days post-encapsulation (Fig. 9). On day 3, the BioLVM spheres had the highest content of DNA among the alginates, e.g. 2.7-fold higher than with LVG spheres (*P* > 0.05) (Fig. 9). After 21 days, the highest decreases in the DNA contents were observed in spheres with high mannuronic acid content (LVM, BioLVM, and BioMVM), although only significant differences were noted with LVM (*P* < 0.001). The DNA contents of the cells embedded in alginate spheres with high guluronic acid content (LVG, BioLVG, and BioMVG) significantly increased from day 3 (*P* < 0.001). An histomorphometrical analysis of PCNA-positive cells on day 21 revealed a higher proliferation of cells embedded in BioLVM and BioMVG (*P* < 0.001).

**Figure 10:**
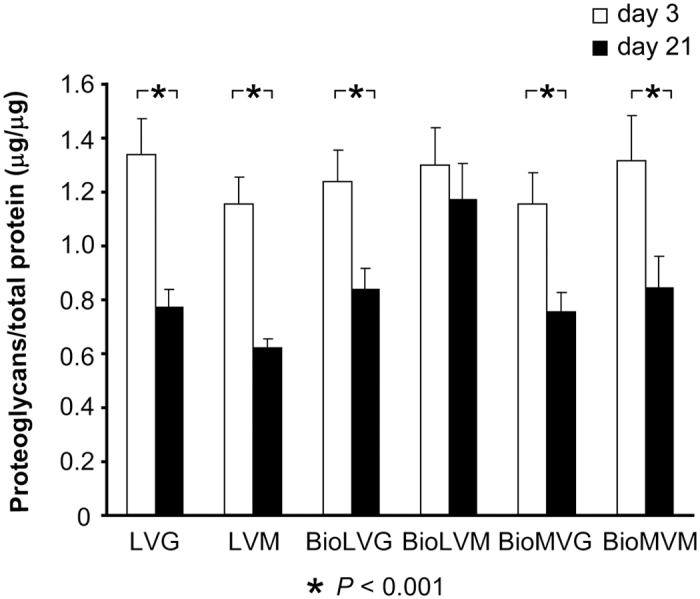
Proteoglycan contents of human meniscal fibrochondrocytes encapsulated in alginate spheres standardized to the total protein contents on day 3 and day 21 (* indicates *P* ≤ 0.001).

**Figure 11:**
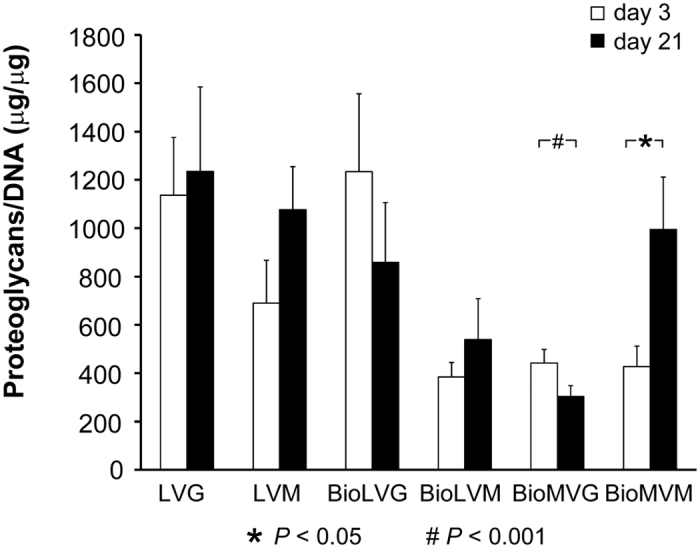
Proteoglycan contents of human meniscal fibrochondrocytes encapsulated in alginate spheres standardized to the DNA contents on day 3 and day 21 (* and # indicate *P* ≤ 0.05 and *P* ≤ 0.01, respectively).

A comparative estimation of the initial proteoglycan contents did not reveal significant differences between alginates (Fig. 10). After 21 days, the contents in the LVG, LVM, BioLVG, BioMVG, and BioMVM spheres were significantly reduced (*P* < 0.001) (Fig. 10). When expressed per DNA on day 3 post-encapsulation, the highest amounts of proteoglycans were detected with BioLVG and LVG alginates (Fig. 11). After 21 days, the amounts of proteoglycans per DNA significantly increased only when human meniscal fibrochondrocytes were embedded in ultrapure medium viscosity high mannuronic acid alginate (BioMVM) (*P* < 0.05). In contrast, the proteoglycans per DNA were significantly reduced over time when cells were cultured in BioMVG (*P* < 0.001) (Fig. 10). No such differences were observed in all other types of alginates.

### A meniscal explant culture model to study the transplantation of alginate spheres containing meniscal fibrochondrocytes into meniscal defects

**Figure 12:**
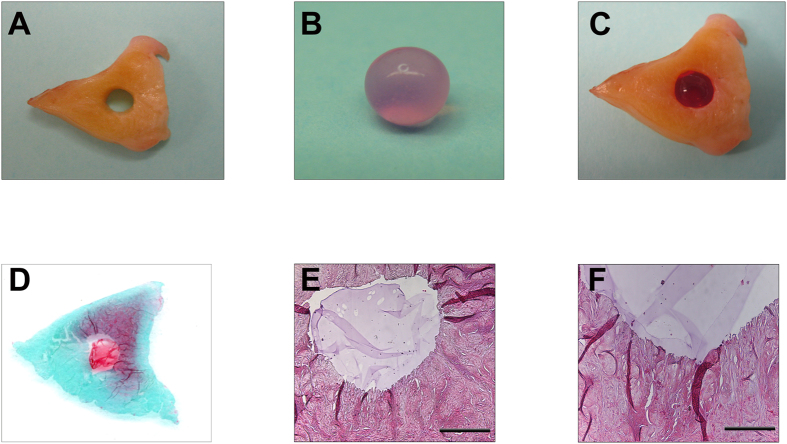
*Ex vivo* transplantation of BioMVM alginate containing human meniscal fibrochondrocytes in an experimental model of meniscal defect. Macroscopic view of (**A)** Meniscal explant culture model with cylindrical defect (**B**) BioMVM alginate sphere containing human meniscal fibrochondrocytes before transplantation into the defect and (**C**) Composite meniscal defect model with a BioMVM alginate sphere. Histological examination of the composite model by (**D**) Safranin O/Fast green staining, (**E**) H&E staining (magnification 2x); scale bar 1,000 μm, and (**F**) H&E staining (magnification 4x); scale bar 500 μm.

The central (inner) parts of the meniscus contain the most proteoglycans, supporting its weightbearing function^31^. As the data revealed that proteoglycan production was optimal with BioMVM, we created a meniscal explant culture model to study the transplantation of alginate spheres containing meniscal fibrochondrocytes into areas where proteoglycan content is of major importance. Transplantation of alginate spheres containing human meniscal fibrochondrocytes was possible, and the spheres remained in place without evidence for loss or fragmentation for 3 days *in vitro*. Histological examination of transverse sections of the composite cultures on day 3 revealed the close proximity of the alginate spheres with the meniscal tissue adjacent to the defect (Fig. 12).

## Discussion

Culture of human meniscal fibrochondrocytes in alginate allows to maintain their physiological state within the hydrogel network and may thus be of high value for cell transplantation approaches. In the present study, we tested the suitability of different alginates to provide the best 3-D microenvironment for human meniscal fibrochondrocytes. First, the data demonstrated that the purity of the alginate affects the shape of the resulting spheres, with spheres based on biomedical-grade alginate with high mannuronic acid content being spherically the most uniform. A decrease in the size of all spheres was noted over time, with biomedical-grade high mannuronic acid content (BioLVM and BioMVM) spheres showing the lowest reduction. The data next indicate that the purity of the alginates does not affect the viability of the encapsulated human meniscal fibrochondrocytes. A significant decrease in the number of viable cells was reported over time in all types of alginates tested being more pronounced in BioLVM and BioLVG alginates. Of note, only cells encapsulated in BioMVM alginate produced and retained significant amounts of proteoglycans per cell, suggesting that BioMVM may be the best suited type of alginate to support proteoglycan production in primary human meniscal fibrochondrocytes in 3-D culture.

The 3-D environment better supports the phenotype and proliferative activities of meniscal fibrochondrocytes compared with monolayer culture^29^. However, specific effects of the 3-D microenvironment upon the ability to maintain their phenotype have been only rarely studied^10^,^29^. Culture of meniscal fibrochondrocytes in alginate spheres increased the synthesis of proteoglycans^32^, cell numbers, and transgene expression of genetically modified cells^33^. In good agreement with previous work^27^,^28^, we observed here a relationship between the size and shape of alginate spheres and the composition and the purity of the alginate used. The shape of the spheres is essential for the functional survival of encapsulated cells^34^,^35^, as fragmented spheres or those containing many satellites are associated with protrusion of cells^36^ and inflammatory responses^37^. Controllable swelling properties are indispensable features of alginate spheres^38^. The use of purified alginates in the present study minimized imperfections and led to more uniform spheres. These results support previous studies reporting a higher shrinkage during gel formation in low guluronic alginate^38^. The decrease of the size of all alginate spheres is in contrast with earlier observations which showed that a softer and less porous structure leads to the disintegration of spheres rich in mannuronic acid residues^38^,^39^ but are in good agreement with other findings^27^ and may be explained by differences in the experimental setup of testing spheres without or with encapsulated cells^35^.

Embedded human meniscal fibrochondrocytes remained viable and metabolically active as previously noted for articular chondrocytes^27^,^28^. Interestingly, the purity of the alginates did not affect the cell viability. These findings are in good agreement with previous work describing a decrease in meniscal cell proliferation over time upon encapsulation in alginate^29^ or agarose hydrogels^40^, although they are in contrast with our previous observations when human articular chondrocytes were encapsulated in the same type of alginates^27^. This reduced cell proliferation rate may thus be attributed to a restriction of cell spreading when meniscal cells are induced to acquire a round morphology within the hydrogel network^40^ due to their dual morphology similar to either fibroblasts or chondrocytes^10^,^29^.

The meniscal proteoglycans in the ECM are chiefly responsible for the viscoelastic compressive properties, a pivotal factor in its shock absorber function^29^,^41^. In addition, they maintain the hydration grade of the tissue forming a basis to provide to the meniscal tissue a high capacity to resist compressive loads through compressive stiffness^29^,^42^. Noteworthy, the central parts of the menisci contain the highest glycosaminoglycan concentrations, besides the meniscal attachments^31^. The present study provides insight into the ability of proteoglycans synthesis by meniscal fibrochondrocytes upon alginate encapsulation. A pattern of production similar to explant cultures was observed, indicating a phenotype resembling the native situation^32^. Interestingly, differential synthesis of proteoglycans was also reported in encapsulated meniscal fibrochondrocytes derived from different meniscal regions^32^. Noticeably, only meniscal fibrochondrocytes embedded BioMVM alginate produced and retained significant amounts of proteoglycans over the 21-day culture period. These data are of high relevance for cell transplantation approaches based on alginates into meniscal areas with a high proteoglycan content.

Finally, the meniscal defect explant culture model employed here may be used to test the effect of different defect and alginate sizes and compositions on cellular behaviour in a relatively natural environment in a standardized and reproducible manner with the view of improving strategies for meniscal repair. For example, interactions of paracrine factors (secreted by the meniscal tissue) with the transplanted fibrochondrocytes within the alginate or *vice versa* (e.g. secreted following gene transfer into meniscal fibrochondrocytes^33^,^43^) may be investigated^33^,^43^. If the defects are of a smaller diameter and filled with constructs allowing for the migration of fibrochondrocytes, mechanisms of cell-based meniscal defect repair could be explored.

A possible limitation of this study is the lack of *in vivo* data. Implantation of selected alginate spheres in experimentally created meniscal lesions in translational minipig^44^ or sheep models^45^,^46^ is the next step that will provide more information about functionality of such hydrogels in a clinically relevant *in vivo* environment. Also, contractile markers determining the response of meniscus to injury such as α-smooth muscle actin (α-SMA) may be studied using this explant culture model, e.g. using human meniscal explants^46^. On the other hand, using meniscal fibrochondrocytes from healthy donors may exclude any influence of possible previous pharmacological treatments such as intra-articular injections of steroids.

Altogether, biomedical-grade, high mannuronic acid content alginate enhanced proteoglycan production of primary human meniscal fibrochondrocytes in 3-D hydrogel culture. These data are of promising value for cell-based translational studies aiming at restoring lost meniscal tissue in regions containing high amounts of proteoglycans.

## Materials and Methods

### Materials

All reagents were purchased at Invitrogen/GIBCO (Karlsruhe, Germany) unless otherwise indicated. L-cystein, Na_2_EDTA, calf thymus DNA, were from Sigma (Munich, Germany). Collagenase type I (activity: 232 U/mg) was purchased at Biochrom (Berlin, Germany). Dimethylmethylene blue was obtained from Serva (Darmstadt, Germany). Chondroitin-6-sulfate from shark cartilage was purchased at Fluka (Munich, Germany). Plasticware was from Falcon (Becton Dickinson, Pont de Claix, France). The PCNA (proliferating cell nuclear antigen) antibody was from Santa Cruz Biotechnology (Heidelberg, Germany). Biotinylated secondary antibody and the ABC reagent were from Vector Laboratories (Alexis Deutschland GmbH, Grünberg, Germany). The investigation was performed at the Center of Experimental Orthopaedics, Saarland University (Saarland, Germany).

### Alginates

Six types of alginates were used in this study^27^,^28^: a nonbiomedical-grade, low viscosity, high mannuronic acid content alginate (LVM) (Sigma), a nonbiomedical-grade, low viscosity, high guluronic acid content alginate (LVG) (Fluka), and four purified and biomedical-grade alginates (Novamatrix, Sandvika, Norway), including a low viscosity, high guluronic acid content alginate (BioLVG), a low viscosity, high mannuronic acid content alginate (BioLVM), a medium viscosity, high guluronic acid content alginate (BioMVG), and a medium viscosity, high mannuronic acid content alginate (BioMVM).

### Isolation and cultivation of human meniscal fibrochondrocytes

Human meniscal fibrochondrocytes were isolated from normal human adult menisci obtained from knee joints treated by total knee arthroplasty from (n = 4, average age 65.5 years ± 13.2, range 46–75 years) as previously described^33^,^43^. The study was approved by the Ethics Committee of the Saarland Physicians Council (Ärztekammer des Saarlandes, Ethik-Kommission, No. 67/12). All patients provided informed consent before inclusion in the study, and all procedures were in accordance with the Helsinki Declaration. Menisci with degeneration on gross examinations were excluded. Menisci were washed, diced into 4 × 4 mm pieces and transferred to DMEM with 100 U/ml penicillin G and 100 μl/ml streptomycin (basal medium) containing 2% fetal bovine serum (FBS) (growth medium) and 0.01% (w/v) collagenase at 37 °C in a humidified atmosphere with 5% CO_2_ for 16 h. Isolated cells were filtered through a 100 μm mesh to remove undigested matrix, washed twice with phosphate-buffered saline (PBS), seeded into 75-cm^2^ tissue culture flasks and kept at 37 °C. Cells were encapsulated in alginate spheres at passage 2.

### Encapsulation of human meniscal fibrochondrocytes in alginate spheres

Cells were trypsinized, washed, and resuspended in sterile-filtered 1.2% alginate in 0.15 M NaCl at a density of 10^6^ cells/ml as previously described^27^,^28^,^33^. The cell suspension was extruded through a 21-gauge needle (Braun, Melsungen, Germany) into 102 mM CaCl_2_ under constant shaking and allowed to polymerize for 10 min. After one wash in PBS and three washes in DMEM, the spheres were maintained at 37 °C in a humidified atmosphere of 5% CO_2_ in growth medium that was changed twice per week (Fig. 1).

### Microscopic evaluation of the alginate spheres

The morphology of the alginate spheres using each type of alginate were estimated on days 3 and 21 post-encapsulation using an inverted optical microscope (CKX-4; Olympus; Hamburg, Germany). The dimensions of the alginate spheres were measured using a computer-based image analysis (n = 4)^27^,^47^,^48^. Images of the whole spheres were acquired by a solid-state CC-12 digital camera (Soft Imaging System) mounted on an inverted microscope analyzed with the analySIS Five Program (Soft Imaging System Corp., Munster, Germany).

### Cell proliferation and viability

On days 0, 1, and 21 after encapsulation, individual spheres were dissolved in 100 μl of 55 mM sodium citrate and 90 mM NaCl (pH 6.8) for 20 min at room temperature. Meniscal fibrochondrocytes were counted using a Neubauer chamber and cell viability was determined by trypan blue exclusion (n = 4)^28^. Parallel cell viability within the spheres at 3 and 21 days post-encapsulation was qualitatively assessed using fluorescence staining with propidium iodide and acridine orange using a fluorescence microscope (Olympus CKX41). After removing the medium spheres were washed carefully with PBS twice and incubated with acridine orange/propidium iodide staining solution (10 μg/ml each in PBS) for 10 min at room temperature^49^,^50^. Live cells are visualized in green (acridine orange) *versus* dead cells in red (propidium iodide).

### Total sulphated glycosaminoglycan content of alginate spheres

On days 3 and 21 post-encapsulation, alginate spheres were dissolved as described above and the released meniscal fibrochondrocytes were incubated overnight in 125 μg/ml papain in 1x PBE (100 mM sodium phosphate buffer, 10 mM Na_2_EDTA, pH 6.5)^28^. Proteoglycans were measured spectrophotometrically by binding to the dimethylmethylene blue dye using chondroitin-6-sulfate to generate a standard curve (n = 6)^51^,^52^. The DNA contents were determined by the Hoechst 33258 assay^53^,^54^,^55^. All data were normalized to the total protein contents assessed by a BCA assay (Pierce, Limburg, Germany), and to the DNA contents^53^,^54^,^55^. All measurements were performed on a GENios spectrophotometer/fluorometer (Tecan, Crailsheim, Germany).

### Histological and immunohistochemical evaluation of the alginate spheres

Spheres based on the different alginates with encapsulated meniscal fibrochondrocytes (n = 4) were harvested after 3 an 21 days of *in vitro* culture, fixed in 4% buffered formalin, dehydrated in graded alcohols, and embedded in paraffin^53^,^54^,^55^. Paraffin-embedded sections (5 μm) were stained with hematoxylin and eosin (H&E) and safranin O according to routine protocols^56^. Expression of PCNA was detected by immunohistochemistry using a specific primary antibody as described elsewere^53^,^54^,^55^. To control for secondary immunoglobulins, sections were processed with omission of the primary antibody. Samples were examined under light microscopy (Olympus BX 45). The percentage of cells positive for PCNA immunostaining was measured using eight serial histological and immunohistochemical sections for each parameter, test, and replicate condition^53^,^54^,^55^.

### Transplantation of alginate spheres containing human meniscal fibrochondrocytes in a meniscal explant culture model

Human menisci were obtained from normal human adult menisci from knee joints treated by total knee arthroplasty (n = 5), and maintained in growth culture medium. Sections of medial menisci (approximately 5-mm thick) were generated using a #21 scalpel blade. Using a 2-mm diameter dermal biopsy punch (Kai Medical, Tokyo, Japan) that was applied strictly perpendicular to the cut meniscal surface, one cylindrical meniscal defect was created in the central (inner) parts of each meniscal section, and one BioMVM alginate sphere containing meniscal fibrochondrocytes was transplanted in a press-fit fashion into the defect and cultured for 3 days in growth medium at 37 °C and 5% CO_2_ (Fig. 1). Composite cultures were then subjected to macroscopic and histological examination with H&E and safranin O/fast green staining^46^,^56^.

### Statistical analysis

Data are given as mean ± standard of the mean (SEM). OneWay ANOVA was used in multiple comparisons. The Student’s t-test was used to detect significant differences when two groups were compared. *P* values of < 0.05 were considered significant. Analyses were conducted using Origin 8 (OriginLab Corporation, Northampton, MA, USA).

## Additional Information

**How to cite this article**: Rey-Rico, A. *et al.* Biomedical-grade, high mannuronic acid content (BioMVM) alginate enhances the proteoglycan production of primary human meniscal fibrochondrocytes in a 3-D microenvironment. *Sci. Rep.* **6**, 28170; doi: 10.1038/srep28170 (2016).
